# *PADI2* Polymorphisms Are Significantly Associated With Rheumatoid Arthritis, Autoantibodies Serologic Status and Joint Damage in Women from Southern Mexico

**DOI:** 10.3389/fimmu.2021.718246

**Published:** 2021-08-04

**Authors:** Iris Paola Guzmán-Guzmán, Claudia Isabel Ramírez-Vélez, Ramcés Falfán-Valencia, José Eduardo Navarro-Zarza, Ilse Adriana Gutiérrez-Pérez, Oscar Zaragoza-García, Mónica Ramírez, Natividad Castro-Alarcón, Isela Parra-Rojas

**Affiliations:** ^1^Faculty of Chemical-Biological Sciences, Universidad Autónoma de Guerrero, Chilpancingo, Mexico; ^2^HLA Laboratory, Instituto Nacional de Enfermedades Respiratorias Ismael Cosío Villegas, Mexico City, Mexico; ^3^Department of Rheumatology and Internal Medicine, Hospital General de Chilpancingo Dr. Raymundo Abarca Alarcón, Guerrero, Mexico; ^4^Consejo Nacional de Ciencia y Tecnología, Universidad Autónoma de Guerrero, Chilpancingo, Mexico

**Keywords:** PADI2, polymorphisms, autoantibodies, radiologic damage, rheumatoid arthritis

## Abstract

The enzymes of the family peptidylarginine deiminases (PADs) have an important role in the pathogenesis of rheumatoid arthritis (RA) due to their association with the anti-citrullinated protein antibodies (ACPA) production. To evaluate the association between single-nucleotide polymorphisms (SNPs) in the *PADI2* gene and RA susceptibility, related clinical parameters, and the serologic status of autoantibodies in a women population with RA from southern Mexico, a case-control study was conducted (case n=229; control n=333). Sociodemographic characteristics were evaluated, along with clinical parameters, inflammation markers, the levels of ACPAs as anti-cyclic citrullinated peptides (anti-CCPs), anti-modified citrullinated vimentin (anti-MCV), and rheumatoid factor (RF). Genomic DNA was extracted from peripheral blood, and three SNPs of the *PADI2* gene (rs1005753, rs2057094, and rs2235926) were performed by qPCR using TaqMan probes. The data analysis reveals that the carriers of the T allele for rs2057094 and rs2235926 presented an earlier onset of the disease (β= -3.26; *p* = 0.03 and β = -4.13; *p* = 0.015, respectively) while the carriers of the T allele for rs1005753 presented higher levels of anti-CCPs (β= 68.3; *p* = 0.015). Additionally, the T allele of rs2235926 was associated with a positive RF (OR = 2.90; *p* = 0.04), anti-MCV (OR = 2.92; *p* = 0.05), and with the serologic status anti-CCP+/anti-MCV+ (OR = 3.02; *p* = 0.03), and anti-CCP+/anti-MCV+/RF+ (OR = 3.79; *p* = 0.004). The haplotypes GTT (OR =1.52; *p* = 0.027) and TTT (OR = 1.32; *p* = 0.025) were associated with the presence of RA. In addition, in this study the haplotype TTT is linked to the presence of radiographic joint damage defined by a Sharp-van der Heijde score (SHS) ≥2 (OR = 1.97; *p* = 0.0021) and SHS ≥3 (OR = 1.94; *p* = 0.011). The haplotype TTT of SNPs rs1005753, rs2057094, and rs2235926 of the *PADI2* gene confers genetic susceptibility to RA and radiographic joint damage in women from southern Mexico. The evidence reveals that SNPs of the *PADI2* gene favors the presence of a positive serologic status in multiple autoantibodies and the clinical manifestations of RA at an early onset age.

## Introduction

Rheumatoid arthritis (RA) is an autoimmune disease with a variable prevalence amongst populations (1, 2). Clinical manifestations for RA can be from a mild self-limiting arthritis to a progressive multisystemic inflammatory arthritis with high morbidity and mortality (3, 4). In the RA physiopathology, enzymes of family peptidylarginine deiminases (PADs) have an important role in the citrullination of proteins that promote the antibody synthesis against citrullinated proteins (ACPAs) (5–8). The ACPAs positive status has been described as a predictive marker for the severity, the radiological degree (9, 10), joint damage, and the functional disability in patients with RA (11, 12). The growth of ACPAs aimed against citrullinated proteins’ epitopes –histones, vimentin and enolase-derived peptides– and fibrinogen is mainly identified during pre-clinical stages of RA. Furthermore, the levels of ACPAs are correlated to the increase of proinflammatory cytokines (TNF-α, IL-6, IL-12p70, IFN-γ, IL-2, and IL-15) and high sensitivity C-reactive protein (hsCRP) (13). In addition, it has been stated that its positive status, along with the rheumatoid factor (RF), promotes the inflammatory process in RA (14).

The expression of the PAD2 and PAD4 isoenzymes have been identified in synovial tissue and fluid in RA patients (15, 16). Although PADs exhibit a limited expression in some tissues, PAD2 is considered the broader expressed isoform (17), and the specificity of its substratum could be related to the clinic phenotype and serologic variability observed in RA patients. It was determined that PAD2 possesses specificity against β/γ-actin, myelin basic protein, histones (H3R26), vimentin, and the glial fibrillary acidic protein (6, 18–21). Meanwhile, for PAD4, the main protein substrates are histones (H2A, H3R2, H3R8, H3R17, H3R26, and H4R3), nuclear lamin C, nucleophosmin/B23, p300/CBP, p21, and inhibitor of growth 4 (6, 22–27). Particularly, high levels of PAD2 have been reported in synovial fluid of RA patients (28–31), as well as their correlation with inflammation markers, the disease’s clinical activity, and the anti-CCPs levels (32).

On the other hand, the influence of genetic factors on the modulation of the expression and function of PADs has been proven (8), and, even though the role of single-nucleotide polymorphisms (SNPs) and of a functional haplotype in *PADI4* has been established, few studies that have determined the role of SNPs in *PADI2* in the genetic susceptibility to RA or its association with the serologic status and the clinical parameters that are related to the disease. Lee et al. reported that *PADI2* could be a candidate-like gene for RA (33) and, in some populations, it was determined that SNPs rs2076596 (34), rs1005753 (35), rs2057094, and rs2235926 (29) in the *PADI2* gene are associated with the presence of RA.

This study aims to evaluate the association between SNPs, rs1005753, rs2057094, and rs2235926 of the *PADI2* gene and RA susceptibility as the relation with the clinical parameters, inflammation markers, and the serologic status for antibodies in a women population from southern Mexico.

## Materials and Methods

### Subject Selection

A case-control study was carried out in RA patients (*n* = 229 women). These patients were diagnosed with RA according to the ACR/EULAR criteria 2010 (36). The control group (*n* = 333 women) was treated for external causes to RA, autoimmune disease, musculoskeletal disease, or cancer in the General Hospital Dr. Raymundo Abarca Alarcón, in Chilpancingo, in the state of Guerrero, Mexico. The case and control subjects were recruited during the period from December 2017 to December 2019. The study was approved by the Research Ethics Committee of the Autonomous University of Guerrero, Mexico (approval code CB-004/2017). All patients agreed to participate and gave their informed consent in writing.

### Clinical Assessment

All of the patients were surveyed to obtain sociodemographic data. The clinical and treatment characteristics were evaluated during the consultation and from the clinical file. In this study “Patients of recent diagnostic” refers to those patients without pharmacological prescription for anti-rheumatic treatment to the date of the sample obtainment.

The rheumatologist performed a clinical evaluation and counted the number of inflamed and painful joints. The patient indicated the level of perception of health status and level of pain perception through a visual analog scale. Rheumatoid arthritis disease activity and the disability level were evaluated through the Disease Activity Score 28 (DAS28), and the Spanish version of the Health Assessment Questionnaire (HAQ-DI), respectively.

To determine joint damage, a radiographic evaluation was performed to observe features of destructive and proliferative changes, and radiological damage. According to Sharp-van der Heijde Score (SHS), this method reviews plain films of 8 proximal interphalangeal joints, 2 interphalangeal thumb joints, 10 metacarpophalangeal joints, and both wrists. The method defines 5 categories: 0 =  normal; 1 =  asymmetrical or minimal narrowing up to a maximum of 25%; 2 =  definite narrowing with loss of up to 50% of the normal space; 3 =  definite narrowing with loss of 50-99% of the normal space or subluxation; and, 4 =  absence of joint space, presumptive evidence of ankylosis, or complete luxation (37).

### Assay of RA-Related Antibodies and Inflammation Markers

Using a venous blood sample, the erythrocyte sedimentation rate (ESR) was analyzed by the Wintrobe method. Serum samples were used to determine the levels of high sensitivity C- reactive protein (hsCRP) and rheumatoid factor (RF) isotype IgG, using the immunoturbidimetry technique in the automatized reader (COBAS C311, Roche Diagnostics GmbH, Germany). In addition, the anti-cyclic citrullinated peptide (anti-CCPs) and anti-modified citrullinated vimentin (anti-MCV) autoantibodies were measured with a second-generation ELISA kit (DIASTAT anti-CCP Axis-Shield, Dundee, United Kindom; and ORGENTEC Diagnostika GmbH, Mainz, Germany, respectively). Values >20 UI/mL were considered positive for RF, values >5 U/mL for anti-CCPs, and >20 U/mL for anti-MCV, according to the manufacturer instructions.

### DNA Extraction and TaqMan Genotyping

Genomic DNA was extracted from peripheral blood and the SNPs were genotyped by allele discrimination using commercial TaqMan probes (Applied Biosystems, San Francisco, CA, USA). The evaluated SNPs were C_2190445_20 (rs1005753/Intron, cat. 4351379), C_11647256_20 (rs2057094/Intron, Cat.4351379), and C_2190476_1_ (rs2235926/3´UTR, Cat.4351379). These were evaluated using quantitative polymerase chain reaction (qPCR) in a 7300 Real-Time PCR System (Applied Biosystems/Thermo Fisher Scientific Inc., Singapore), following the instructions by the manufacturer. The thermal cycling was performed by denaturation at 60°C for 30 sec, followed by 40 cycles of 95°C for 10 min and 95°C for 15 sec, and alignment and extension at 60°C 1 min and 4°C. Genotype analysis was performed through the sequence detection software (SDS) version 2.3 (Applied Biosystems, CA, United States).

### Statistical Analysis

**Figure 1:**
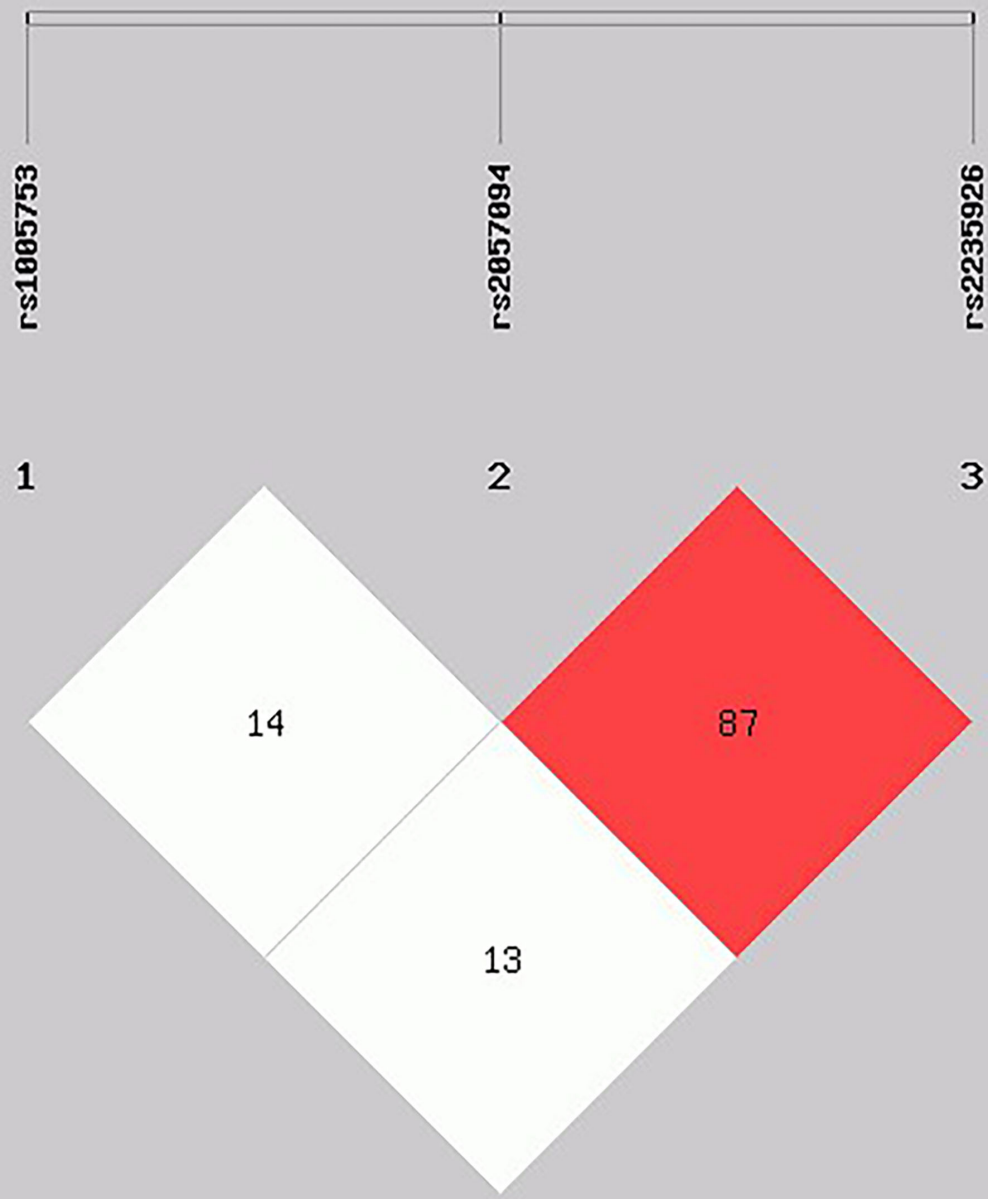
Linkage disequilibrium (LD) test of *PADI2* gene SNPs in RA patients. Haplotype frequencies and LD were calculated using SHEsis software. Red area represents higher levels of LD. A D´ value of 100 indicates a complete LD between two markers and a D´ value of 0 indicates complete linkage equilibrium.

The categorical variables were expressed as numbers and proportions, and they were compared using a Chi-squared test. For quantitative variables, median and percentiles p5-p95th were used according to the Mann Whitney test to compare groups. Allele and genotype frequencies were calculated by direct counting. The differences in the distributions of allele and genotype frequencies between cases and controls, and the associations between clinical characteristics in patients with RA, were performed using a Chi-square test and a logistic regression. The Hardy-Weinberg equilibrium (HWE) was assessed in both groups. The effect of polymorphisms on clinical assessment and serum autoantibodies levels and other clinical parameters was tested using β coefficient and their standard errors, adjusted by age, treatment with disease-modifying anti-rheumatic drugs (DMARDs) and wood smoke exposure. Statistical analysis was carried out using the Stata version 13.0 (StataCorp, College Station, TX, USA). The association and pairwise measure of linkage disequilibrium of the SNPs rs1005753, rs2057094, and rs2235926 of PADI2 was calculated using SHEsis software (38) (**Figure 1**). The association between SNPs and clinical parameters and serologic status was determined using logistic regression models, settling odds ratios (ORs), and 95% confidence intervals (95% CI). Results were considered significant at *p <*0.05.

## Results

### Demographic Data, Clinical Features, and Serologic Status

The sociodemographic and clinical characteristics between the case and control groups are presented in **Table 1**. The participants with RA displayed a period of evolution of the disease with a median of 7 years, along with a low functional disability HAQ-DI score. At the same time, they also presented a moderate clinical activity and a radiographic joint damage index that refers to the presence of the narrowing of the intra-articular space and bone erosion, even though most of them were pharmacologically treated with DMARDs. Furthermore, the autoantibodies’ levels (RF, anti-CCPs, and anti-MCV) were elevated. When comparing the positive serologic status to the antibodies, more than 85% of the subjects were positive, with a predominant positive status for anti-MCV (89.1%). After evaluating the serologic status of combined positivity, it was found that 83.8% were anti-CCPs+/anti-MCV+ and that 75.1% were positive for ACPAs and RF (anti-CCP+/anti-MCV+/RF+) (**Table 2**).

**Table 1:** Demographics and clinical characteristics in the study population.

Characteristics	RA *n* = 229	CS *n* = 333	*p-*value
**Sociodemographic features**			
Age, years, median (P_5-_P_95_)^a^	44 (24-70)	50 (25-71)	0.003
Socioeconomic level, *n* (%)^b^			0.59
Medium	51 (22.3)	68 (20.2)	–
Low	178 (77.7)	265 (79.8)	–
Wood smoke exposure, *n* (%)^b^	139 (60.7)	138 (41.4)	<0.001
Smoking, *n* (%)^b^	6 (2.62)	15 (4.5)	0.24
**Serological features**			
RF, IU/mL, median (P_5-_P_95_)^a^	182 (0-306)		
Anti-CCP, U/mL, median (P_5-_P_95_)^a^	50.8 (0.25-243)		
Anti-MCV, U/mL, median (P_5-_P_95_)^a^	83.5 (14-620)		
**Clinical assessment**			
Disease evolution, years, median (P_5-_P_95_)^a^	7 (1-26)	–	–
hsCRP, mg/L, median (P_5-_P_95_)^a^	7 (0.16-60.2)	–	–
ESR, mm/hr, median (P_5-_P_95_)^a^	31 (9-56)	33 (13-55)	0.03
DAS28-ESR, score, median (P_5-_P_95_)^a^	3.71 (1.99-7.67)	–	–
DAS28-ESR, *n* (%)^b^			
Remission	40 (17.5)	–	–
Low activity	52 (22.8)	–	–
Moderate activity	73 (31.6)	–	–
High activity	64 (28.1)	–	–
HAQ-DI, score, median (P_5-_P_95_)^a^	0.3 (0.-1.52)	–	–
SHS, median (P_5-_P_95_)^a^	2 (1-4)	–	–
**Current therapy scheme** ^b^			
Recent diagnostic, *n* (%)^b^	66 (28.8)	–	–
Monotherapy DMARDs, *n* (%)^b^	40 (17.5)	–	–
Combination DMARDs, *n* (%)^b^	123 (53.7)	–	–

Anti-CCPs, anti-cyclic citrullinated peptide antibodies; Anti-MCV, Antibodies against modified citrullinate vimentin, CS, control subjects; DAS28, disease activity score 28; DMARDs, disease-modifying antirheumatic drugs; ESR, erythrocyte sedimentation rate; HAQ-DI, health assessment questionnaire disability index; hsCRP, high sensibility protein c reactive; RA, rheumatoid arthritis; RF, rheumatoid factor; SHS, Sharp-van der Heijde Score.

a Data are expressed as the median and percentiles 5th-95th.

b Data are expressed as the n (%), compared using Chi-square test.

p-value < 0.05 was considered statistically significant.

**Table 2:** Serologic pattern status in RA patients.

Characteristics	*n* = 229
**Positive antibodies**	
RF+, >20 IU/mL, *n* (%)	199 (86.9)
Anti-CCP+, >5 U/mL, *n* (%)	198 (86.45)
Anti-MCV+, > 20 U/mL, *n* (%)	204 (89.1)
**ACPA status**	
Anti-CCP+/Anti-MCV+, *n* (%)	192 (83.84)
Anti-CCP-/Anti-MCV-, *n* (%)	19 (8.3)
Anti-CCP+/Anti-MCV-, *n* (%)	6 (2.62)
Anti-CCP-/Anti-MCV+, *n* (%)	12 (5.24)
**ACPA/RF status**	
Anti-CCP+/Anti-MCV+/RF+, *n* (%)	172 (75.1)
Anti-CCP+/Anti-MCV+/RF-, *n* (%)	20 (8.73)
Anti-CCP-/Anti-MCV+/RF+, *n* (%)	12 (5.24)
Anti-CCP-/Anti- MCV-/RF+, *n* (%)	11 (4.8)
Anti-CCP-/Anti-MCV-/RF-, *n* (%)	8 (3.5)
Anti-CCP+/Anti-MCV-/RF+, *n* (%)	4 (1.75)
Anti-CCP+/Anti-MCV-/RF-, *n* (%)	2 (0.87)

ACPAs, anti-citrullinated protein antibodies; Anti-CCPs, anti-cyclic citrullinated peptide antibodies; Anti-MCV, Antibodies against modified citrullinate vimentin; RA, rheumatoid arthritis; RF, rheumatoid factor.

Data are expressed as the n (%).

### Frequency of *PADI2* Gene SNPs and Haplotype Analysis

The genotypical distribution of the SNPs rs1005753 (T>G), rs2057094 (T>C), and rs2235926 (T>C) of the *PADI2* gene was in Hardy-Weinberg genetic equilibrium in both studied groups (*p* > 0.5). The genotypical and allele frequencies are shown in **Table 3**. The T allele of rs2057094 was marginally associated with RA susceptibility (OR = 1.27; 95% CI, 0.99-1.65; *p* = 0.05), however, the haplotypes TTT (OR=1.32; 95% CI, 1.03-1.67; *p* = 0.025) and GTT (OR = 1.52; 95% CI, 1.04-2.19; *p* = 0.027) were significantly associated with RA susceptibility (**Table 4**).

**Table 3:** Genotypic and allele frequencies of polymorphisms in *PADI2* gene.

SNP	RA *n* = 229	CS *n* = 333	OR (95% CI), *p-*value
rs1005753			
TT, *n* (%)	126 (55.0)	195 (58.7)	1.0*
TG, *n* (%)	88 (38.4)	121 (36.3)	1.12 (0.78-1.6), 0.51
GG, *n* (%)	15 (6.6)	17 (5.1)	1.36 (0.65-2.8). 0.40
Allele			
T, *n* (%)	340 (74.2)	511 (76.7)	1.0*
G, *n* (%)	118 (25.8)	155 (23.3)	1.14 (0.86-1.52), 0.33
HWE X^2^, *p-value*	X^2^ = 0.005; *p* = 0.94	X^2^ = 0.10; *p* = 0.75	
rs2057094			
TT, *n* (%)	106 (46.3)	120 (36.0)	1.0*
TC, *n* (%)	93 (40.6)	166 (49.9)	0.63 (0.44-0.91), **0.014**
CC, *n* (%)	30 (13.1)	47 (14.1)	0.72 (0.42-1.22), 0.22
Allele			
T, *n* (%)	305 (66.6)	406 (61.0)	1.0*
C, *n* (%)	153 (33.4)	260 (39.0)	0.78 (0.60-1.0), **0.05**
HWE X^2^, *p-value*	X^2^ = 1.74; *p* = 0.18	X^2^ = 0.74; *p* = 0.38	
rs2235926			
TT, *n* (%)	100 (43.7)	135 (40.5)	1.0*
TC, *n* (%)	107 (46.7)	156 (46.9)	0.92 (0.65-1.32), 0.67
CC, *n* (%)	22 (9.6)	42 (12.6)	0.70 (0.39-1.25), 0.23
Allele			
T, *n* (%)	307 (67.03)	426 (63.96)	1.0*
C, *n* (%)	151 (32.97)	240 (36.04)	0.87 (0.67-1.13), 0.28
HWE X^2^, *p-value*	X^2^ = 0.74; *p =* 0.38	X^2^ = 0.09, *p =* 0.76	

CI, confidence interval CS, control subjects; HWE, Hardy-Weinberg Equilibrium; OR, odds ratio; RA, rheumatoid arthritis; SNP, single-nucleotide polymorphism.

Data n (%), compared using Chi-square test. Logistic regression calculated OR and 95% CI. *1.0 Reference category. p values were calculated by logistic regression comparisons with the refence category.

p-value < 0.05 was considered statistically significant.

Bold values represent statistically significant data.

**Table 4:** Haplotype frequencies of three *PADI2* SNPs in RA and CS.

Haplotypes	RA *n* = 229 *n* (%)	CS *n* = 333 *n* (%)	OR (95% CI), *p*-value
H1: 111 TTT	229.83 (50.0)	288.77 (0.43)	1.32 (1.03-1.67), **0.025**
H2: 112 TTC	5.62 (0.012)	42.96 (0.064)	0.18 (0.07-0.43), **0.00002**
H3: 121 TCT	6.72 (0.015)	36.52 (0.055)	0.26 (0.11-0.58), **0.0005**
H4: 122 TCC	97.83 (0.21)	142.74 (0.21)	0.99 (0.74-1.32), 0.96
H5: 211 GTT	62.79 (0.13)	63.11 (0.09)	1.52 (1.04-2.19), **0.027**
H6: 212 GTC	6.75 (0.01)	11.16 (0.01)	ND
H7: 221 GCT	7.66 (0.01)	37.6 (0.05)	0.28 (0.12-0.62), **0.0008**
H8: 222 GCC	40.79 (0.09)	43.14 (0.06)	1.41 (0.90-2.20), 0.13

CI, confidence interval; CS, control subjects; H, haplotype; ND, not determinate; OR, odds ratio; RA, rheumatoid arthritis; SNPs, single-nucleotide polymorphisms.

The SNPs are listed in the order: PADI2 rs1005753_T>G, rs2057094_T>C and rs2235926_T>C. The OR and 95% CI and p values were obtained by SHESIS test.

p-value < 0.05 was considered statistically significant.

Bold values represent statistically significant data.

### Association of Clinical Parameters and Serologic Status According to the SNPs and Haplotype of the *PADI2* Gene in RA Patients

In this study, we evaluated the potential effect and the association between *PADI2*’s SNPs and clinical parameters, inflammation markers, and the serologic status in opposition to autoantibodies in RA patients. The presence of the T allele of SNPs rs2057094 and rs2235926 was associated with the presence of the disease’s clinical signs at a younger age (β = -3.26; *p* = 0.03 and β = -4.13; *p* = 0.02, years respectively), while the presence of the T allele of rs1005753 was associated with the increase of the anti-CCPs levels (β = 68.3; *p* = 0.01) (**Table 5**).

**Table 5:** Effect of *PADI2* SNPs on the clinical characteristics in RA patients.

Characteristics	rs1005753		rs2057094		rs2235926	
	^†^GG *vs* TT+TG β 95% CI,* p-*value	^†^TT *vs* GG+TG β 95% CI, *p-*value	^†^CC *vs* TT+TC β 95% CI, *p-*value	^†^TT *vs* CC+TC β 95% CI, *p-*value	^†^CC *vs* TT+TC β 95% CI, *p-*value	^†^TT *vs* CC+TC β 95% CI, *p-*value
Age at diagnosis, years	-1.45 (-5.49, 2.59), 0.48	2.11 (0.12, 4.11), **0.04**	-3.26 (-6.2, -0.32), **0.03**	0.32 (-1.67, 2.32), 0.75	-4.13 (-7.46, -0.81), **0.02**	0.04 (-1.96, 2.06), 0.96
ESR, mm/hr	2.43 (-5.49, 10.35), 0.54	1.24 (-2.68, 5.16), 0.53	1.94 (-3.85, 7.75), 0.50	2.18 (-1.72, 6.9), 0.27	3.39 (-3.18, 9.97), 0.31	1.89 (-2.04, 5.83), 0.34
DAS28-ESR, score	0.47 (-0.37, 1.33), 0.27	0.18 (-0.24, 0.61), 0.39	0.16 (-0.47, 0.80), 0.61	0.31 (-0.10, 0.74), 0.14	0.09 (-0.63, 0.82), 0.80	0.28 (-0.14, 0.71), 0.18
HAQ-DI, score	0.20 (-0.07, 0.48), 0.15	-0.05 (-0.19, 0.09), 0.48	0.13 (-0.07, 0.34), 0.19	0.04 (-0.10, 0.17), 0.60	0.15 (-0.08, 0.39), 0.18	0.02 (-0.11, 0.16), 0.74
Morning stiffness, min	3.55 (-44.23, 50.3), 0.88	-3.13 (-26.4, 20.14), 0.79	0.04 (-0.12, 0.21), 0.61	21.0 (-2.01, 44.0), 0.07	11.0 (-27.9, 49.9), 0.57	-0.06 (-0.18, 0.05), 0.28
SHS	0.14 (-0.50, 0.80), 0.65	-0.35 (-0.68, -0.025), **0.03**	-0.07 (-0.56, 0.42), 0.78	-0.07 (-0.41, 0.25), 0.63	-0.36 (-0.92, 0.20), 0.20	-0.11 (-0.45, 0.22), 0.50
RF, IU/mL	8.35 (-119.7, 136.4), 0.89	15.4 (-50.6, 81.42), 0.64	44.0 (-47.4, 135.4), 0.34	-3.08 (-16.34, 10.2), 0.64	88.8 (-14.9, 192.6), 0.09	7.71 (-57.4, 72.9), 0.81
Anti-CCP, U/mL	68.3 (16.3, 120.4), **0.01**	-10.0 (-37.0, 16.86), 0.46	1.0 (-35.8, 37.8), 0.95	-6.76 (-33.7, 20.2), 0.62	11.14 (-30.8, 53.1), 0.60	3.94 (-23.0, 30.9), 0.77
Anti-MCV, U/mL	35.54 (-80.0, 151.1), 0.54	-37.85 (-96.6, 20.9), 0.20	0.74 (-44.2, 84.0), 0.98	-7.08 (-66.8, 52.67), 0.81	-2.06 (-92.4, 88.2), 0.96	6.03 (-53.9, 65.9), 0.84

Anti-CCPs, anti-cyclic citrullinated peptide antibodies; Anti-MCV, Antibodies against modified citrullinate vimentin; CI, confidence interval; DAS28, disease activity score 28; DMARDs, disease-modifying antirheumatic drugs; ESR, erythrocyte sedimentation rate; HAQ-DI, health assessment questionnaire disability index; RA, rheumatoid arthritis; RF, rheumatoid factor. SHS, Sharp/van der Heijde Score; SNP, single-nucleotide polymorphism.

β, regression coefficient and 95% CI. Model adjusted by age, DMARDs treatment and wood smoke exposure.

^†^Reference category. p-value < 0.05 was considered statistically significant.

Bold values represent statistically significant data.

When analyzing the association between SNPs and the autoantibodies’ serologic status, the carriers of the T allele of SNP rs2057094 exhibited a tendency to associate with the presence of positive RF (OR = 2.5; 95% CI, 0.93-6.6; *p* = 0.06) and with the positive status for ACPAs and RF (anti-CCP+/anti-MCV+/RF+, OR = 2.23; 95% CI, 0.96-5.18; *p* = 0.06). In addition, the genotypes TT+TC of rs2235926 were related to the individual positivity to RF (OR = 2.90; 95% CI, 1.02-8.26; *p* = 0.04), and to anti-MCV (OR = 2.92; 95% CI, 0.96-8.9; *p* = 0.05), as well as with the positive status for anti-CCP+/anti-MCV+ (OR = 3.02; 95% CI, 1.11-8.23; *p* = 0.03) and anti-CCP+/anti-MCV+/RF+ (OR = 3.79; 95% CI, 1.51-9.5; *p* = 0.004) (**Table 6**).

**Table 6:** Association of *PADI2* SNPs on the clinical characteristics in RA patients.

Characteristics	rs1005753		rs2057094		rs2235926	
	^†^GG *vs* TT+TG OR 95% CI, *p-*value	^†^TT *vs* GG+TG OR 95% CI, *p-*value	^†^CC *vs* TT+TC OR 95% CI, *p-*value	^†^TT *vs* CC+TC OR 95% CI, *p-*value	^†^CC *vs* TT+TC OR 95% CI, *p-*value	^†^TT *vs* CC+TC OR 95% CI, *p-*value
Age at diagnosis, <40 years	0.62 (0.08-4.76), 0.65	1.42 (0.58-3.46), 0.44	1.17 (0.31-4.37), 0.80	1.62 (0.67-3.95), 0.28	1.73 (0.39-7.67), 0.46	1.67 (0.68-4.1), 0.25
DAS28-ESR, >3.2 Units	1.77 (0.57-5.4), 0.31	0.87 (0.49-1.55), 0.65	1.05 (0.46-2.4), 0.89	1.19 (0.67-2.12), 0.54	1.5 (0.57-3.9), 0.40	1.31 (0.73-2.34), 0.35
DAS28-ESR, >5.1 Units	1.91 (0.21-16.8), 0.55	0.76 (0.31-1.87), 0.56	2.32 (0.54-9.8), 0.25	1.30 (0.53-3.2), 0.56	2.11 (0.45-9.9), 0.34	1.40 (0.59-3.47), 0.46
HAQ-DI, 1-2 Units	1.78 (0.35-8.9), 0.48	0.86 (0.42-1.76), 0.69	1.64 (0.44-6.04), 0.45	1.38 (0.67-2.8), 0.37	5.46 (0.52-11.61), 0.25	1.21 (0.59-2.46), 0.59
Radiologic score, SHS >2	3.92 (0.76-20.2), 0.10	0.40 (0.20-0.81), **0.01**	0.84 (0.29-2.38), 0.74	0.64 (0.32-1.30), 0.22	0.33 (0.08-1.34), 0.12	0.61 (0.30-1.25), 0.18
Radiologic score, SHS >3	1.44 (0.41-5.1), 0.56	0.39 (0.19-0.82), 0.01	0.74 (0.26-2.15), 0.59	0.93 (0.96-1.92), 0.86	0.31 (0.07-1.26), 0.10	0.80 (0.39-1.0), 0.55
RF+ (>20 IU/mL)	0.70 (0.13-3.54), 0.67	1.16 (0.52-2.54), 0.71	2.5 (0.93-6.6), 0.06	1.23 (0.56-2.67), 0.60	2.90 (1.02-8.26), **0.04**	1.17 (0.53-2.55), 0.69
Anti-CCP+ (>5 U/mL)	1.81 (0.45-7.2), 0.39	0.98 (0.45-2.12), 0.96	1.07 (0.33-3.4), 0.90	1.51 (0.70-3.27), 0.28	1.55 (0.48-5.0), 0.45	1.67 (0.77-3.61), 0.19
Anti-MCV+ (>20 U/mL)	1.22 (0.25-5.9), 0.80	0.60 (0.26-1.40), 0.24	1.52 (0.47-4.9), 0.48	1.86 (0.79-4.36), 0.15	2.92 (0.96-8.9), **0.05**	2.05 (0.87-4.8), 0.10
Anti-CCP+/Anti-MCV+	1.43 (0.36-5.59), 0.60	0.83 (0.40-1.69), 0.61	1.60 (0.69-4.4), 0.35	1.43 (0.70-2.98), 0.32	3.02 (1.11-8.23), **0.03**	1.59 (0.78-3.26), 0.20
Anti-CCP+/Anti-MCV+/FR+	0.91 (0.26-3.14), 0.88	1.21 (0.65-2.26), 0.52	2.23 (0.96-5.18), 0.06	1.29 (0.70-2.38), 0.40	3.79 (1.51-9.5), **0.004**	1.32 (0.72-2.43), 0.36

Anti-CCPs, anti-cyclic citrullinated peptide antibodies; Anti-MCV, Antibodies against modified citrullinate vimentin; CI, confidential interval; DAS28, disease activity score 28; DMARDs, disease-modifying antirheumatic drugs; ESR, erythrocyte sedimentation rate; HAQ-DI, health assessment questionnaire disability index; RA, rheumatoid arthritis; RF, rheumatoid factor. SHS, Sharp/van der Heijde Score; SNP, single-nucleotide polymorphism.

OR, odds ratio and 95% CI. Model adjusted by age, DMARDs treatment and wood smoke exposure.

^†^Reference category. p-value < 0.05 was considered statistically significant.

Bold values represent statistically significant data.

Finally, in the association analysis among haplotypes of *PADI2* gene and the clinical characteristics of the disease, the haplotype TTT was significantly related with the presence of joint damage defined by a SHS score ≥ 2 (OR = 1.97; 95% CI, 1.27-3.05; *p* = 0.002) and SHS ≥3 (OR = 1.94; 95% CI, 1.15-3.19; *p* = 0.011).

## Discussion

Our finds prove the potential role of *PADI2* single-nucleotide variants in the clinical heterogeneity of RA in a women population of southern Mexico. Chang et al. studied a Chinese population and proved that SNPs rs2235926 (OR = 1.57; *p <*0.001) and rs2057094 (OR = 1.36; *p* = 0.003) conferred RA susceptibility (29). In our study, these SNPs were not associated individually with RA, but they were associated with the presence of high levels of anti-CCPs and a positive status for anti-CCPs+/anti-MCV+/RF+. In the *PADI4* gene, the presence of SNPs and a functional haplotype has been described as a factor for genetic susceptibility to RA in different populations, including the Mexican (39–41). In the Mexican population, the GTG haplotype in *PADI4* was associated with the RA emergence in ages ≤40 years old and with elevated anti-CCPs levels (40), as well as with anti-MCV antibodies, which are related with the increase of inflammatory cytokines levels and the RA DAS28 score (42). In this study, we found that the T allele of rs2235926 and rs2057094 in *PADI2* was associated with the rise of RA in ages younger than 40 years old. Even though the age of onset for RA is variable, in the Mexican population, the highest point of most incidence fluctuates between 56-65 years old (2), similar to what was described in a Chinese population where it fluctuates between 60-70 years of age (43). It has been described that the citrullination process precedes the appearance of the disease’s clinical signs (44, 45), therefore the genetic susceptibility attributed to the *PADIs* genes involved in the protein citrullination process and of its interaction with other genetic and environmental factors could determine the clinical appearance of RA in ages younger than the largest incidence peak of the disease.

Particularly for our population, the observed seropositivity for ACPAs (>80%), as well as the combined positivity for anti-CCP+/anti-MCV+RF+ (75.1%) is high, especially when compared to that described in other populations where seropositivity oscillates between 38% and 53.9% (42, 46). Anti-CCPs positivity is considered a predictive marker for structural joint damage in RA patients (47), and for higher radiographic and inflammation progression (48–50). In this study, the TTT haplotype of the studied SNPs of the *PADI2* gene was associated with a representative score for joint damage, and particularly, the SNP rs2235926 with the ACPAs positivity (anti-CCPs+/anti-MCV+) and ACPAs/RF (anti-CCPs+/anti-MCV+/RF+). Furthermore, the seropositivity to multiple autoantibodies of the ACPAs type and RF isotopes is associated with the radiological progression and erosive RA (51), just as with the high levels of C-reactive protein (CRP) and proinflammatory cytokines in RA patients (13, 52–54). In an *in vitro* model, it was proven that the RF from isotype IgM favors the increase of the anti-CCPs levels and the cytokine production, thus suggesting that a positive serologic status for RF and anti-CCPs contributes to the pathogenesis of RA (14), as well as to the clinical diversification of the disease (12). On the other hand, the anti-MCV antibodies are considered better predictors of the disease’s severity (55), high clinical activity, and radiographic joint damage and progression (53, 56), given that it increases up to 7.3 times the risk of radiographic progression (10) when compared to anti-CCPs. In our study, the common allele of SNP rs2235926 was associated with the positivity for anti-MCV.

The peptide repertoire that is susceptible to citrullination by PAD2 is more extensive in comparison to PAD4. Assohou-Luty et al. characterized 320 citrullination sites for PAD2 and 178 sites for PAD4, thus proving the specificity variability of these enzymes to their substrate (57). Two main regions have been described in PADs that are involved in the substratum selection, the N-terminal domain, and the cleft of the active site. In PAD4, the Arg-374 contributes to recognizing the substratum and its structural formation, while in PAD2 and PAD3, the amino acid is Gly-374 (58). In this study, we found a relation between positivity to anti-CCPs, anti-MCV and RF, and SNP rs2235926 of *PADI2*. This polymorphism is found in a 3´untranslated region, however, even though it hasn’t been defined, this could influence the post-transcriptional expression of PAD2 and the citrullination of peptides. Nevertheless, PAD2 can citrullinate transcript factors that determine the Th0 cell lineage differentiation, such as GATA3 and RORγt. Citrullination of R300 of GATA3 and 4 arginine residues (R56, R59, R77, and R90) from RORγt, proves it has an effect on the gene regulation and on cell functions (59). This is important because the presence of polymorphisms in *PADI2* could modulate the expression and enzymatic function, therefore relating not only with the serologic positivity to a wide repertoire of antibodies, but also with the activation of naive T cells in response to antigen or “self” antigens, and their subsequent proliferation and differentiation, involved in the pathogenesis and clinical diversification of RA.

Conversely, the enzymatic activity of PADs is mediated by the Ca^2+^ concentrations. In PAD4 five binding sites for Ca^2+^ have been identified. The binding of this element to their sites induces conformational changes that create the active site cleft of the enzyme (60), while in PAD2, the Ca^2+^ binding happens in 6 sites. The activation of the binding sites 3, 4, and 5 control the dependency on Ca^2+^ and on regulatory elements that include a Ca^2+^ switch (61). The citrullination by PAD2 and PAD4 requires Ca^2+^ concentrations in a range of 0.35 to 1.85 mM (30). Vossenar et al. reported that in samples of synovial fluid from RA patients that vimentin can be citrullinated by PAD2 and PAD4 isotypes (28). However, using mass spectrometry, it was found that of 47 sites in fibrinogen that are susceptible to citrullination, 46 of them were citrullinated by PAD2 *in vitro* (62). Furthermore, PAD2 could have more protein citrullination activity such as fibrin, and also it could increase the proinflammatory cytokines’ expression (63), indicating the leading role of PAD2 in the pathogenesis of RA related not only with the production of ACPAs but also with the modulation of the inflammatory process. We consider that the identification of the susceptibility polymorphisms in *PADI2* is important from the clinical viewpoint; however, the clinical perspective of our findings related to the presence of a positive serologic status for multiple autoantibodies and the clinical manifestations of RA at an early onset age deserves replication studies amongst populations.

Citrullination of vimentin in joints is crucial during the pathogenesis of RA (64). In an *in vitro* model, it was observed that citrullinated vimentin increases the secretion of TNF-α and IL-1, as well as the expression of PAD4 and RANKL (65). Moreover, during the differentiation process from monocyte to macrophage, the mRNA and PAD2 protein levels increase (66, 67). Likewise, it was observed that the expression of the mRNA of *PADI2* is high in CD34^+^ cells and that its expression levels are correlated to the Sp1 transcription factor (68), which, interestingly, along with transcription factor Sp3 has an influence over the *PADI2* transcription (69). This proves the important role of PAD2 in the regulation of the expression of proinflammatory cytokines, of genes that promote citrullination, and of differentiation processes and activation of cells involved in joint bone resorption. In this study the haplotype TTT was significantly related with the presence of joint damage defined by a SHS, however other studies are required to clarify the functional role of SNPs in the *PADI2* gene on expression of the protein.

It was proven that the expression of PAD2 is elevated in samples of synovial tissue from RA patients (28–31). Damagaard et al. reported that the levels of PAD2 were high in synovial fluid in RA and that these are higher in patients that are positive to anti-CCPs. Additionally, the PAD2 levels in synovial fluid correlated with clinical activity, the levels of CRP, anti-CCPs, and leukocyte count, as well as cytokines such as IL-6, IL-8, and IL-10 (32). In an *in vitro* model, it was observed that PAD2 could promote IL-1β, IL-6, and TNF-α production in macrophages, and apoptosis induction when activating the caspases 2, 3, and 9, and, at the same time, when activating cell adhesion by FAK, paxillin, and PAK1 ways, which leads to the increase of inflammation (70). Meanwhile, the presence of anti-PAD2 antibodies is associated with a small number of inflamed joints, with a low prevalence of interstitial lung disease, and slower progression of joint damage (71). However, the current study is not exempt from limitations, including the lack of validation in a second population. Moreover, other studies are required to clarify the functional role of SNPs in the *PADI2* gene.

In conclusion, in a women population from southern Mexico, the TTT haplotype in the *PADI2* gene confers genetic susceptibility to RA and radiographic joint damage related to a positive status to autoantibodies anti-CCP+/anti-MCV+/RF+ and the clinical manifestations of RA at an early onset age.

## Data Availability Statement

The original contributions presented in the study are included in the article/supplementary material. Further inquiries can be directed to the corresponding author.

## Ethics Statement

The studies involving human participants were reviewed and approved by CB-004/2017. The patients/participants provided their written informed consent to participate in this study.

## Author Contributions

IG-G Designed the study and wrote the manuscript. OZ-G, CR-V, and JN-Z recruited the patients, control subjects, and obtained the samples. IG-P and OZ-G data collection. CR-V, IG-P, and RF-V performed the experiments. MR, NC-A, and IP-R provided relevant opinions in the manuscript. All authors contributed to the article and approved the submitted version.

## Conflict of Interest

The authors declare that the research was conducted in the absence of any commercial or financial relationships that could be construed as a potential conflict of interest.

## Publisher’s Note

All claims expressed in this article are solely those of the authors and do not necessarily represent those of their affiliated organizations, or those of the publisher, the editors and the reviewers. Any product that may be evaluated in this article, or claim that may be made by its manufacturer, is not guaranteed or endorsed by the publisher.
